# Dystrophic phenotype improvement in the diaphragm muscle of *mdx* mice by diacerhein

**DOI:** 10.1371/journal.pone.0182449

**Published:** 2017-08-07

**Authors:** Rafael Dias Mâncio, Túlio de Almeida Hermes, Aline Barbosa Macedo, Daniela Sayuri Mizobuti, Ian Feller Rupcic, Elaine Minatel

**Affiliations:** Department of Structural and Functional Biology, Institute of Biology, State University of Campinas (UNICAMP), Campinas, São Paulo, Brazil; University of Minnesota Medical Center, UNITED STATES

## Abstract

Chronic inflammation and oxidative stress are striking features of Duchenne muscular dystrophy disease. Diacerhein is an anthraquinone, which exhibits anti-inflammatory and antioxidant properties. Based on their actions, the present study evaluated the effects of diacerhein against myonecrosis, oxidative stress and inflammatory response in the diaphragm muscle of *mdx* mice and compared these results to current treatment widely used in DMD patients, with a main focus on the impact of prednisone. The results demonstrated that diacerhein treatment prevented muscle damage indicated by a decrease in the IgG uptake by muscle fibers, lower CK levels in serum, reduction of fibers with central nuclei with a concomitant increase in fibers with peripheral nuclei. It also had an effect on the inflammatory process, decreasing the inflammatory area, macrophage staining and TNF-α and IL-1β content. Regarding oxidative stress, diacerhein treatment was effective in reducing the ROS and lipid peroxidation in the diaphragm muscle from *mdx* mice. Compared to prednisone treatment, our findings demonstrated that diacerhein treatment improved the dystrophic phenotype in the diaphragm muscle of *mdx* mice similar to that of glucocorticoid therapy. In this respect, this work suggests that diacerhein has a potential use as an alternative drug in dystrophinopathy treatment and recommends that its anti-inflammatory and antioxidants properties in the dystrophic muscle should be better understood.

## Introduction

Duchenne muscular dystrophy (DMD) is a lethal and X-linked muscle disease characterized by cycles of muscle degeneration and regeneration, chronic inflammation and an oxidative stress state, which affects 1 in 3500–6000 live male birth [1–4].

Regarding the inflammatory process, it has been reported that inflammatory cytokines such as tumor necrosis factor α (TNF-α) and interleukin-1β (IL-1β) play a major role in the DMD phenotype [5]. In *mdx* mice, the experimental model of DMD [6], the TNF-α content has been well characterized and shown to have increased in the diaphragm muscle where inflammation is generally greater in this model [7–9]. The IL-1β also contributes towards muscular dystrophy, leading to the initiation and continuation of the muscle pathology in DMD [10]. Previous studies have shown that blocking the TNF-α and IL-1β pathways improves the dystrophy phenotype in *mdx* mice [11–13].

Oxidative stress, as mentioned above, is a hallmark feature of DMD disease. Dystrophic patients and *mdx* mice showed high levels of reactive oxygen species (ROS) and lipid peroxidation [5, 14]. Our research group and other researchers have already demonstrated the beneficial effect of antioxidant treatment, such as N-acetylcysteine, ascorbic acid, green tea and taurine, in dystrophic muscles [8, 15–17].

Diacerhein (1,8-diacetoxy-9,10-dioxo-dihydroanthracene-3-carboxylic acid) is an anthraquinone normally used in osteoarthritis treatment, which exhibits antiinflammatory properties [18]. Rhein, its active metabolite, has been shown to inhibit TNF-α, IL-6 and IL-1β synthesis and activity, particularly the IL-1β proinflammatory cytokine [19]. Besides this, diacerhein is known to reduce the production of superoxide anion, negatively modulate the synthase and activity of inducible nitric oxide synthase and may also inhibit IKKβ activity [20, 21].

Based on the anti-inflammatory and antioxidant effects of diacerhein, this drug has a capacity to improve the dystrophic muscle pathology and has an established safety profile after many years of use for osteoarthritis. So, in this study, we evaluated the anti-inflammatory and antioxidant properties of diacerhein against myonecrosis, oxidative stress and inflammatory response in the diaphragm muscle of *mdx* mice.

## Methods

### Animals characterization

All experiments described here were carried out based on the guidelines of the Brazilian College for Animal Experimentation (COBEA) and approved by the Ethics Committee of our institution (process n° 3334–1). Newborn male and female C57BL/10 (C57BL/10ScCr/PasUnib) and *mdx* (C57BL/10-Dmd*mdx*/PasUnib) mice were maintained on a regimen of 12-h dark, 12-h light cycles, room temperature at 21°C and were weaned at 21 days of age. After this period, they had *ad libitum* access to food and water. The *mdx* mice were randomly divided into three groups: *mdx* (untreated group), *mdx*P (prednisone-treated group) and *mdx*D (diacerhein-treated group). The C57BL/10 mice were designated as a control group (Ctrl).

### Diacerhein and prednisone-administration protocol

*Mdx* mice (14 days old) received diacerhein 20mg/kg/day (TRB Pharma Indústria Química e Farmacêutica Ltda®, São Paulo, Brazil) or prednisone 5mg/kg/day (EMS®, São Paulo, Brazil) by oral gavage, diluted in 0.05 ml saline for 14 days. Diacerhein and prednisone treatments were initiated in *mdx* mice before the onset of muscle degeneration-regeneration cycles (from about 14–17 days) [22] to verify the effects on the early stage of the disease. In order to accurately adjust the drug dose, each animal was weighed daily. Control *mdx* mice (14 days old) and C57BL/10 mice (14 days old) received saline only for 14 days and were used as controls.

### Grip strength testing

Forelimb muscle strength was evaluated with a grip strength meter (New Primer, São Paulo, Brazil), as previously described [9, 23]. Five measurements were obtained for each animal from each experimental group at the beginning (14 days old) and end (28 days old) of the time period. Absolute strength was normalized to body weight at 14 and 28 days. N = 5 for each group analyzed.

### Blood samples for creatine kinase and IL-1β, IL-6 analysis

The animals (n = 5 for each group analyzed) were anaesthetized using a mixture of ketamine hydrochloride (130 mg/kg; Francotar, Virbac, Fort Worth, TX, USA) and xylazine hydrochloride (6.8 mg/kg, 2% Virbaxil; Virbac), and blood samples (0.8 ml) were taken for biochemical assessment of muscle fiber degeneration and inflammatory cytokine, as previously described [9, 23].

#### Creatine kinase assay

The samples were microcentrifuged at 3000 rpm for 10 min and the supernatant (serum) was removed and used for analysis after incubation at room temperature for 1–2 h to allow for clotting. The creatine kinase (CK) assay was carried out using a commercially available kit (CK Cinético Crystal, BioClin, Ireland) and a BioTek Spectrophotometer (BioTek Instruments Inc., Winooski, VT, USA). Values are reported as international units per litre.

*Enzyme-linked immunosorbent assay* *(ELISA)*

The samples were quantified using a Quantikine IL-1β ELISA kit (Sigma-Aldrich, St. Louis, MO, USA) according to the manufacturer’s instructions.

### Histopathological analysis

#### Degeneration/regeneration process evaluation (n = 5 for each group analyzed)

For morphological visualization and quantification of muscle fiber damage the diaphragm (DIA) and tibialis anterior (TA) muscles, cryosections were incubated with fluorescently labelled immunoglobulin (Ig) G. In brief, muscle cryosections (8 μm thick) were preincubated for 30 min with 5% bovine serum albumin (BSA) in phosphate-buffered saline (PBS), followed by a 1 h incubation with IgG fluorescein isothiocyanate conjugate antibody (anti-mouse; Sigma-Aldrich, St Louis, MO, USA) [9]. The number of IgG-positive muscle fibers was expressed as a percentage of the total number of muscles fibers counted in each section (4–5 sections per muscle) from all experimental groups.

For morphological analysis of regenerated muscle fibers (central nucleated fibers) and mature fibers (peripheral nucleated fibers), cryosections of the DIA and TA muscles were stained with hematoxylin/eosin. Slides were examined under a Nikon Eclipse TS100 microscope connected to a computer and a video camera (Nikon DS-Ri1). Non overlapping images of the entire cross-section were taken and tiled together using the NIS-elements AR Advances Research software (Nikon Instruments Inc., Melville, NY, USA) [24]. The number of central nucleated fibers and fibers with peripheral nuclei, expressed as a percentage of the total number of fibers, was determined in each cross-section (4–5 sections per muscle).

#### Inflammation evaluation (n = 5 for each group analyzed)

Muscle inflammation was measured by hematoxylin and eosin staining and F4/80 immunohistochemistry, as previously described [9].

Inflammatory cells were identified in hematoxylin and eosin-stained sections in terms of nucleus morphology and cell size, and showed basophilic nuclear staining and little cytoplasm. Areas containing densely packed inflammatory cells were measured using the NIS-elements AR Advances Research software.

Muscle sections for F4/80 staining were fixed in acetone for 10 min, air dried for 20 min and washed with PBS. Sections were washed with PBS and blocked for 1 h at room temperature with 3% BSA in PBS. The slides were incubated overnight at 4°C with primary antibody against F4/80 (monoclonal antibody; AbD Serotec, Raleigh, NC, USA). After PBS washes, the slides were incubated with anti-rat secondary antibody (Texas Red® Anti-rat IgG; Vector Laboratories, Burlingame, CA, USA) for 1 h at room temperature. After washing with PBS, the muscle sections were mounted in 1,4-diazabicyclo[2.2.2]octane (DABCO; Sigma) mounting medium for fluorescence microscopy (Nikon Eclipse TS100). F4/80 staining was quantified using NIS-elements AR Advances Research software.

For hematoxylin and eosin staining and F4/80 immunofluorescence the percentage of total muscle area was calculated in each section studied (four or five sections per muscle). A blinded observer carried out the counts and measurements.

#### Oxidative stress evaluation (n = 5 for each group analyzed)

Oxidative stress was analyzed by counting the number of autofluorescent granules of lipofuscin and dihydroethidium (DHE) reaction.

Cryostat unfixed transverse sections, 8-μm-thick, of the DIA and TA muscles were used for measuring the autofluorescent granules of lipofuscin. The section was mounted in medium for fluorescence microscopy (Nikon Eclipse TS100) and used within 3 days after mounting for the fluorescence measurements. The total number of autofluorescent lipofuscin granules and tissue area were determined in each cross-section (4–5 sections per muscle) from all experimental groups, using the NIS-elements AR Advances Research software. Assuming that the muscle sections were uniformly 8-*μ*m thick, the number of autofluorescent lipofuscin granules per mm^3^ of DIA and TA muscles was calculated for each mouse [25].

To determine the levels of ROS, DIA and TA cross-sections were incubated with DHE, as previously reported [9, 26]. Briefly, the DIA and TA sections were incubated with 5 *μ*l DHE in PBS at 37°C for 30 minutes, and then washed with PBS and mounted in DABCO (Sigma). DHE staining shows as a bright red emission on fluorescence microscopy. The intensity of reactive DHE by muscle area was quantified by measuring pixels in a specific range (70–255 wavelength), which was adjusted to eliminate interference from background fluorescence using the NIS-elements AR Advances Research software.

### Western blot analysis

The NF-κB, TNF-α and 4-hydroxynonenal (4-HNE) content in the DIA and TA muscles of all experimental groups (n = 5 for each group analyzed) was analyzed using Western blotting, as previously reported [9, 23]. An assay lysis buffer containing freshly added protease and phosphatase inhibitors (1% Triton, 10 mM sodium pyrophosphate, 100 mM NaF, 10 *μ*g/ml aprotinin, 1 mM phenylmethanesulphonyl fluoride and 0.25 mM Na_3_VO_4_) was used to lyse the muscles. The samples were centrifuged at 11,000 rpm for 20 min, and the soluble fraction was resuspended in 50 *μ*l Laemmli loading buffer (2% sodium dodecyl sulphate [SDS], 20% glycerol, 0.04 mg/ml bromophenol blue, 0.12 M Tris-HCl, [pH 6.8] and 0.28 M β-mercaptoethanol). A total of 30 *μ*g total protein homogenate from each sample was placed onto 12–15% SDS-polyacrylamide gels. Proteins were transferred from the gels to a nitrocellulose membrane using a submersion electrotransfer apparatus (Bio-Rad Laboratories, Hercules, CA, USA). Membranes were blocked for 2 h at room temperature with 5% skim milk/Tris-HCl buffer saline-Tween buffer (TBST; 10 mM Tris-HCl [pH 8], 150 mM NaCl and 0.05% Tween 20). The membranes were incubated with the primary antibodies overnight at 4°C, washed in TBST, incubated with the peroxidase-conjugated secondary antibodies for 2 h at room temperature and developed using the SuperSignal West Pico Chemiluminescent Substrate kit (Pierce Biotechnology, Rockford, IL, USA). To control for protein loading, Western blot transfer and nonspecific changes in protein levels, the blots were stripped and re-probed for glyceraldehyde-3-phosphate dehydrogenase (GAPDH). Band intensities were quantified using the GeneTools software (SynGene–A Divison of Synoptics, Cambridge, England).

The following primary antibodies were used for Western blotting: (1) NF-κB (goat polyclonal, Santa Cruz Biotechnology, Santa Cruz, California); (2) TNF-α (rabbit anti-mouse polyclonal antibody; Millipore, CA, USA); (3) 4-HNE (goat polyclonal antibody; Santa Cruz Biotechnology); and (4) GAPDH (rabbit polyclonal antibody; Santa Cruz Biotechnology). The secondary antibody was peroxidase-labelled affinity-purified mouse or rabbit IgG antibody (KPL, Gaithersburg, MD, USA).

### Statistical analysis

All data are expressed as mean ± standard deviation (SD). Statistical analysis for direct comparison between means of groups was performed by ANOVA, followed by Tukey test used for multiple statistical comparisons between groups. P≤0.05 was considered statistically significant.

## Results

The control (C57BL/10) group and saline, prednisone- and diacerhein-treated *mdx* groups presented weight gain during the experimental period and there was no significant difference between them (Table 1). All experimental groups showed an increase in muscle strength over the study period, but the saline-treated *mdx* mice had the lowest gain and statistically differed from the control group. Treatment with diacerhein presented the best strength gain in the period, which was significantly different from the saline and prednisone-treated *mdx* groups (Table 1).

**Table 1:** Body weights and forelimb muscle strength in control and dystrophic mice.

	Body weight (g)		Body weight gain/period	Force/Body weitght (g/g)		Force gain / period	
	Start	Final	(%)	14 Days	28 Days		(%)
**Ctrl**	6,67±0,62	12,96±1,92	94,22	1,80±0,15	2,68±0,16	48,87	
***mdx***	6,54±0,63	12,34±1,81	88,83	1,85±0,17	2,09±0,09^**a**^	12,55	
***mdx*P**	7,19±0,53	11,84±1,04	64,77	1,87±0,14	2,16±0,50	15,36	
***mdx*D**	6,81±0,87	12,99±2,00	90,75	1,79±0,26	2,82±0,26^**b**^^**,**^^**c**^	57,88	

Body weight (g) was measured at the beginning (start) and after 2 weeks (final) in C57BL/10 mice (Ctrl), saline-treated *mdx* mice (*mdx*), prednisone-treated *mdx* mice (*mdx*P) and diacerhein-treated *mdx* mice (*mdx*D). Forelimb muscle strength was assessed by taking measurements of force at time points 1 and 2, normalized by body weight (g/g). Body weight gain/period: percentage of body weight gain during the treatment period. Force gain/period: percentage of muscular force gain during the treatment period. All values expressed as mean ± standard deviation (SD).

^a^*P* ≤ 0.05 compared with Ctrl mice

^b^*P* ≤ 0.05 compared with *mdx* mice

^c^*P* ≤ 0.05 compared with prednisone-treated *mdx* mice (one-way ANOVA with Tukey’s post-hoc test).

The degeneration and regeneration processes in the dystrophic DIA and TA muscles were evaluated by intracellular fiber staining with IgG antibody, CK levels and by the number of fibers with central and peripheral nuclei.

**Fig 1:**
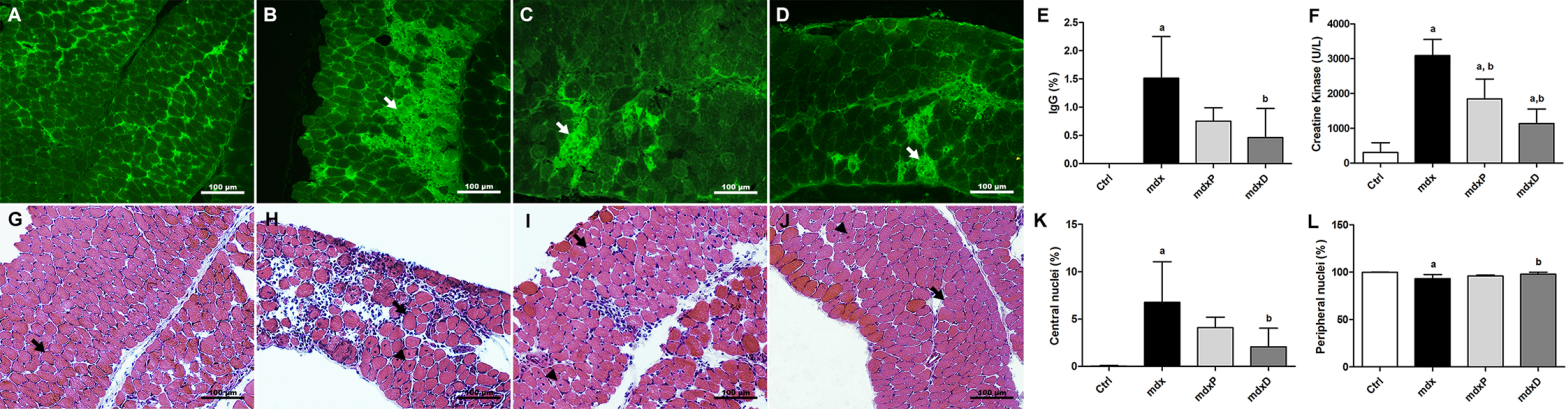
Degeneration/regeneration process in dystrophic diaphragm muscle. Diaphragm (DIA) cross-sections showing IgG staining (white arrow) in C57BL/10 (A), saline-treated *mdx* mice (B), prednisone-treated *mdx* mice (C) and diacerhein-treated *mdx* mice (D). The graphs (E) show the IgG staining (%) in the DIA muscle of C57BL/10 mice (Ctrl), saline-treated *mdx* mice (*mdx*), prednisone-treated *mdx* mice (*mdx*P) and diacerhein-treated *mdx* mice (*mdx*D). The graphs (F) show the creatine kinase (CK) levels in Ctrl, *mdx*, *mdx*P and *mdx*D. CK values expressed as mean ± standard deviation (SD). DIA cross-sections showing fibers which central nuclei (black arrow head) and peripheral nuclei (black arrow) in Ctrl (G), *mdx* (H), *mdx*P (I) and *mdx*D (J). The graphs show the nuclei central fibers (K) and peripheral nuclei fibers (L) in the DIA muscle of Ctrl, *mdx*, *mdx*P and *mdx*D groups. ^a^*P* ≤ 0.05 compared with Ctrl mice, ^b^*P* ≤ 0.05 compared with *mdx* mice (one-way ANOVA with Tukey’s post-hoc test).

The damaged fibers were stained in DIA muscle sections with antibody against IgG. Control (C57BL/10) mice showed no intracellular fiber staining (Fig 1A), while *mdx* mice showed intracellular staining for IgG antibody within some muscle fibers (Fig 1B). The diacerhein-treated *mdx* group showed a significant decrease in IgG staining (69.4%) in the DIA muscle of *mdx* mice (Fig 1D and 1E). Similar results were observed in the TA muscle (S1A–S1E Fig). The biochemical evaluation of muscle fiber degeneration, by CK levels, showed a significant increase of this enzyme in the saline-treated *mdx* mice compared to the control (C57BL/10) mice (Fig 1F). Prednisone- and diacerhein-treated *mdx* groups showed a significant decrease of this increase (40.3% in *mdx*P and 63.2% in *mdx*D) in the diaphragm muscle of *mdx* mice (Fig 1F).

Mature fibers with peripheral nuclei were observed in the diaphragm muscle of C57BL/10 and *mdx* mice (Fig 1G–1J). Regenerated muscle fibers indicated by central nuclei were seen in the dystrophic diaphragm muscle (Fig 1H–1J). The saline-treated *mdx* group showed an expressive number of regenerated muscle fibers and the diacerhein treatment significantly reduced this increase (Fig 1K). A significant decrease of fibers with peripheral nuclei was observed in saline-treated *mdx* mice compared to the control mice (Fig 1L). At the same time, a significant increase of fibers with peripheral nuclei occurred in diacerhein-treated *mdx* mice compared to saline-treated *mdx* mice (Fig 1L). Similar results were observed in the TA muscle (S1F–S1K Fig).

The inflammatory process in the control (C57BL/10) group and saline, prednisone- and diacerhein-treated *mdx* groups was evaluated by determining the inflammatory area; the infiltration of macrophages; and NF-κB, TNF-α, IL-1β and IL-6 content in the DIA and TA muscles.

**Fig 2:**
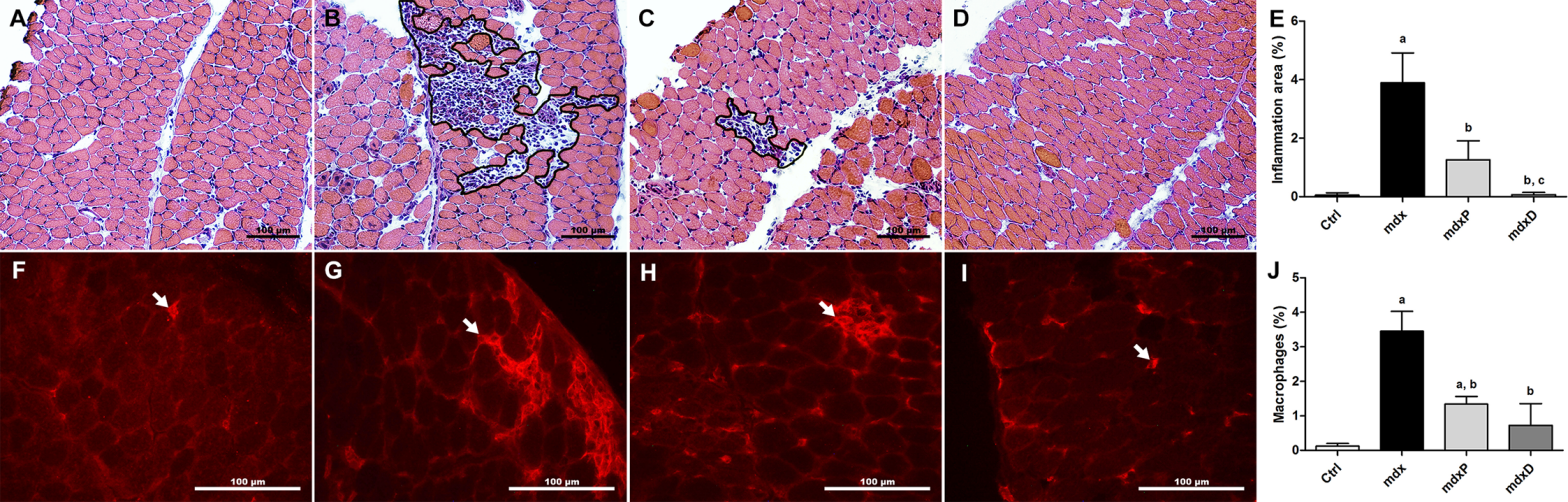
Inflammatory response morphology in dystrophic diaphragm muscle. Diaphragm (DIA) cross-sections showing inflammatory area (outline) in C57BL/10 (A), saline-treated *mdx* mice (B), prednisone-treated *mdx* mice (C) and diacerhein-treated *mdx* mice (D). The graphs (E) show the inflammatory area (%) in the DIA muscle of C57BL/10 mice (Ctrl), saline-treated *mdx* mice (*mdx*), prednisone-treated *mdx* mice (*mdx*P) and diacerhein-treated *mdx* mice (*mdx*D). DIA cross-sections showing F4/80 staining (white arrow) in Ctrl (F), *mdx* (G), *mdx*P (H) and *mdx*D (I). In (J), graphs show the F4/80 staining (%) in the DIA muscle of Ctrl, *mdx*, *mdx*P and *mdx*P groups. ^a^*P* ≤ 0.05 compared with Ctrl mice, ^b^*P* ≤ 0.05 compared with *mdx* mice, ^c^*P* ≤ 0.05 compared with prednisone-treated *mdx* mice (one-way ANOVA with Tukey’s post-hoc test).

The inflammatory area showed conspicuous regions containing inflammatory cells, which were densely packed among regenerated and degenerated muscle fibers (Fig 2B and 2C). Prednisone and diacerhein significantly reduced the area of inflammation (67.6% in *mdx*P and 98.0% in *mdx*D) in the diaphragm muscle of *mdx* mice compared to the saline-treated *mdx* mice, where the diacerhein treatment showed a more expressive reduction (Fig 2E). In terms of the infiltration of macrophages, no macrophages were detected in the diaphragm muscle sections from the control (C57BL/10) mice (Fig 2F and 2J), while *mdx* mice showed a higher proportion of macrophage infiltration (Fig 2G and 2J). Prednisone and diacerhein treatments significantly decreased macrophage infiltration (61.2% in *mdx*P and 78.9% in *mdx*D) compared to the saline-treated *mdx* mice (Fig 2H–2J). Similar results were observed in the TA muscle (S2A–S2J Fig).

**Fig 3:**
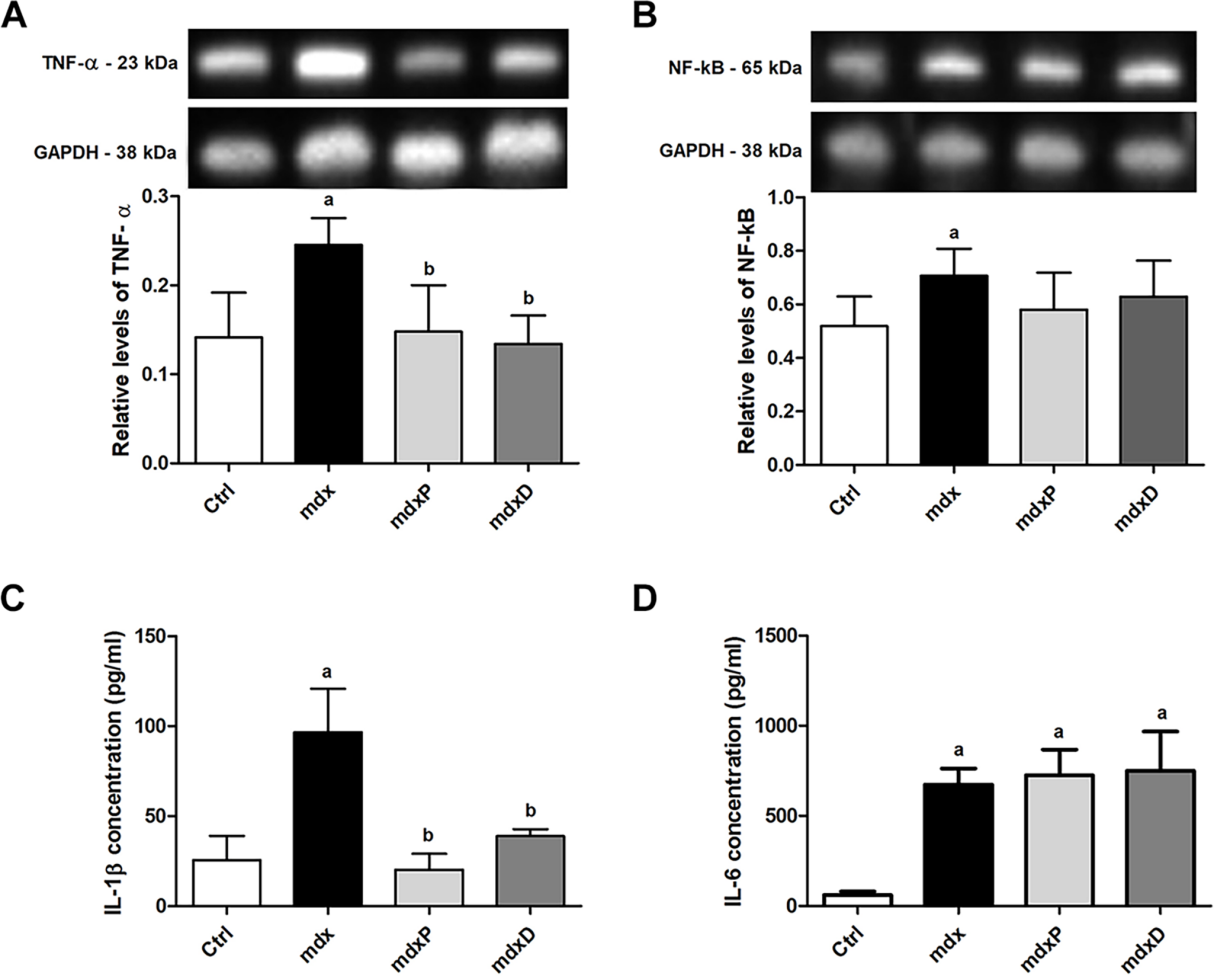
Factor and inflammatory cytokines involved in dystrophic process. Western blotting analysis of tumor necrosis factor alpha (A, TNF-α), and nuclear factor-kappa (B, NF-κB). The blots of the proteins (top row) and of glyceraldehyde-3-phosphate dehydrogenase (loading control, bottom row), are shown. The graphs show protein levels in the crude extracts of diaphragm (DIA) muscle from C57BL/10 mice (Ctrl), saline-treated *mdx* mice (*mdx*), prednisone-treated *mdx* mice (*mdx*P) and diacerhein-treated *mdx* mice (*mdx*D). The intensities of each band were quantified and normalized to those of the corresponding Ctrl. Relative values are expressed as mean ± standard deviation (SD). Detection of IL-1β (C) and IL-6 (D) levels in serum of Ctrl, *mdx*, *mdx*P and *mdx*D groups. ^a^*P* ≤ 0.05 compared with Ctrl mice, ^b^*P* ≤ 0.05 compared with *mdx* mice (one-way ANOVA with Tukey’s post-hoc test).

Immunoblotting revealed a significant increase in TNF-α and NF-κB levels in the saline-treated *mdx* muscle (by 73.6% and 28.7%, respectively) compared to the control (C57BL/10) mice (Fig 3A and 3B). Prednisone and diacerhein treatments significantly reduced the TNF-α level in the DIA muscle (by 39.8% and 45.3%, respectively) in *mdx* mice compared to the saline-treated *mdx* mice (Fig 3A). In the TA muscle the TNF-α and NF-κB levels remained high even after diacerhein treatment (S3A and S3B Fig). Increased IL-1β and IL-6 levels were verified in the saline-treated *mdx* mice (by 277.7% and 977.4%, respectively) compared to the control (C57BL/10) mice (Fig 3C and 3D). Prednisone and diacerhein treatments significantly reduced the IL-1β level (by 74.7% and 59.7%, respectively) in the *mdx* mice compared to the saline-treated *mdx* mice (Fig 3C), but had no effect on IL-6 levels (Fig 3D).

The last analysis performed in the present study was the determination of oxidative stress, which was assessed by DHE reaction, the number of autofluorescent granules of lipofuscin and 4-HNE protein adduct levels.

**Fig 4:**
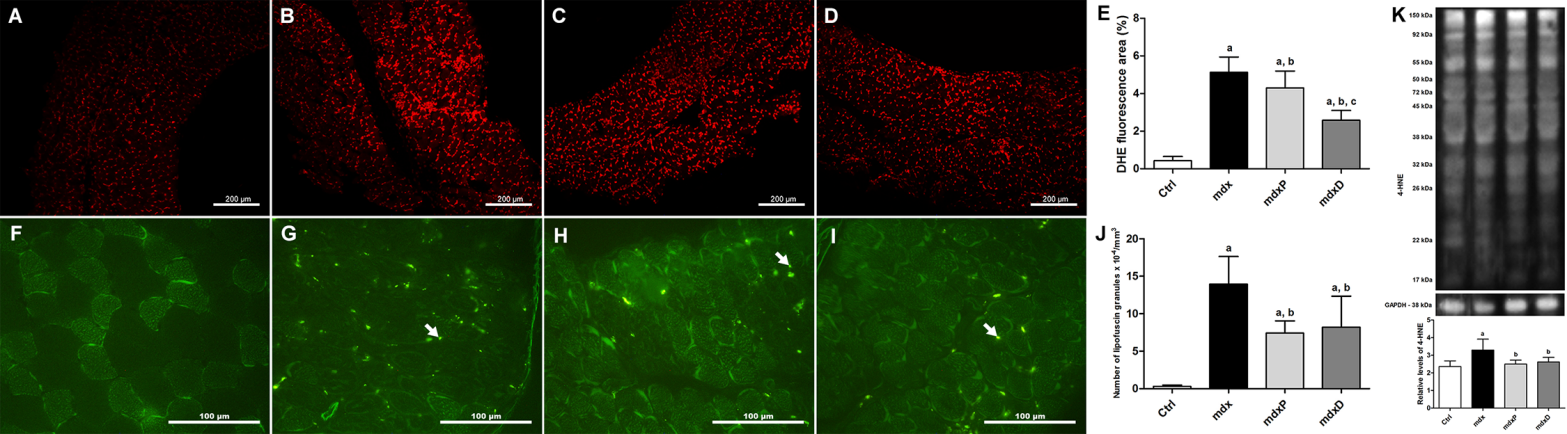
Oxidative stress in dystrophic diaphragm muscle. Diaphragm (DIA) cross-sections showing dihydroethidium (DHE) fluorescence in C57BL/10 (A), saline-treated *mdx* mice (B), prednisone-treated *mdx* mice (C) and diacerhein-treated *mdx* mice (D). The graphs (E) show the DHE staining area (%) in C57BL/10 mice (Ctrl), saline-treated *mdx* mice (*mdx*), prednisone-treated *mdx* mice (*mdx*P) and diacerhein-treated *mdx* mice (*mdx*D). DIA cross-sections showing autofluorescent lipofuscin granules (white arrow) in Ctrl (F), *mdx* (G), *mdx*P (H) and *mdx*D (I). The graphs (J) show the number of lipofuscin granules x 10^−4^/mm^3^ in Ctrl, *mdx*, *mdx*P and *mdx*D groups. In (K), western blotting analysis of 4-hydroxynonenal (4-HNE)-protein adducts. Bands corresponding to protein (top row), and glyceraldehyde-3-phosphate dehydrogenase (GAPDH; used as loading control) (bottom row), are shown. The graphs show protein levels in the crude extracts of DIA muscle from Ctrl, *mdx*, *mdx*P and *mdx*D groups. The intensities of each band were quantified and normalized to those of the corresponding Ctrl (in order to obtain relative values). All values expressed as mean ± standard deviation (SD). ^a^*P* ≤ 0.05 compared with Ctrl mice, ^b^*P* ≤ 0.05 compared with *mdx* mice, ^c^*P* ≤ 0.05 compared with prednisone-treated *mdx* mice (one-way ANOVA with Tukey’s post-hoc test).

The DHE area was significantly (about 74%) larger in saline-treated *mdx* mice compared to the control (C57BL/10) mice (Fig 4A and 4B). Prednisone and diacerhein significantly reduced the DHE area (16.1% in *mdx*P and 49.6% in *mdx*D) in the diaphragm muscle of *mdx* mice compared to the saline-treated *mdx* mice, where the diacerhein treatment showed a more expressive reduction (Fig 4C–4E). Autofluorescent lipofuscin granules in diaphragm muscles from normal and *mdx* mice can be seen in Fig 4F–4I. The lipofuscin granules were expressively pronounced in the diaphragm muscle of saline-treated *mdx* mice compared to the control (C57BL/10) mice (Fig 4J). Prednisone and diacerhein treatments significantly reduced the number of lipofuscin granules (by 46.9% and 41.2%, respectively) in *mdx* mice compared to the saline-treated *mdx* mice (Fig 4J). Similar results regarding DHE area and the number of autofluorescent granules of lipofuscin were observed in TA muscle (S4A–S4J Fig). Bands of 4-HNE-protein adducts are shown in Fig 4K. Proteins from 17 to 150 kDa were observed in all groups. Considering the sum of the all bands (11 bands), the 4-HNE protein adduct levels were significantly higher in saline-treated *mdx* mice (by 39.3%) compared to the control mice (Fig 4K). Reduction in the 4-HNE protein adduct levels was observed in the prednisone- and diacerhein-treated *mdx* groups (by 24% and 20.5%, respectively) compared to the control (C57BL/10) mice (Fig 4K). There was no change in 4-HNE levels between the experimental *mdx* groups in the TA muscle (S4K Fig).

## Discussion

DMD is one of the most prevalent forms of muscular dystrophies for which there is no cure. Prednisone, prednisolone and/or deflazacort are the most commonly used glucocorticoid treatments in this disorder, but exhibit relatively limited therapeutic success and several side effects, such as weight gain, bone demineralization and hypertension [27], indicating that there is a critical need for new pharmacological therapy. In this sense, we evaluated the effects of diacerhein, an established drug used for osteoarthritis with important anti-inflammatory and antioxidant properties [20, 28], in *mdx* mice, focusing on the morphological and molecular alterations of the diaphragm muscle.

Sarcolemmal damage is one of the key early events which leads to the pathological features observed at later stages of this disease progression in muscular dystrophy [29]. The reduction in IgG uptake by muscle fibers and lesser CK levels in the serum of diacerhein-treated *mdx* mice indicate the protective effect of this drug against dystrophic myonecrosis. In addition, this result was reinforced by the reduced number of fibers with central nuclei with a concomitant increase in fibers with peripheral nuclei after diacerhein treatment. Expressive increased forelimb strength in diacerhein-treated *mdx* mice, similar to that observed in normal mice, also demonstrates another positive effect of diacerhein against muscle damage. These diacerhein beneficial effects seen in dystrophic mice may be at least partly justified by the anti-inflammatory action of this drug.

Anti-inflammatory treatments have demonstrated effective effects against myonecrosis in muscular dystrophies. Some of these studies showed that the functional blockade of NF-κB or the inflammatory cytokines, such as TNF-α, IL-6 and IL-1β are a specific therapeutic strategy for the treatment of dystrophinopathies [11, 30–32]. Regarding the anti-inflammatory diacerhein effects in the present work, we observed an expressive reduction in some inflammatory parameters analyzed, which include the inflammatory area, macrophage staining and TNF-α and IL-1β content.

The anti-inflammatory is a most relevant property attributed to diacerhein, previously shown in experimental models [20, 33, 34]. Despite the fact that the precise mechanism of how this drug exerts its anti-inflammatory effects is still not completely understood, several works suggest that diacerhein and its active metabolite, rhein, downregulates the TNF-α, IL-6 and IL-1β expression and inhibits NF-κB activation [20, 35, 36]. However, we did not find a significant reduction in NF-κB and IL-6 levels after diacerhein treatment in *mdx* mice. Perhaps, as many inflammatory pathways are triggered in muscular dystrophy, it is possible that diacerhein, alone, is insufficient to halt the whole process.

Oxidative stress is another important contributor towards muscle damage in muscular dystrophy [37, 38]. Excessive oxidative stress can produce superoxide anions, hydrogen peroxide or nitric oxide. These primary oxidative species are converted to secondary reactive oxygen species (ROS) and reactive nitrogen species (RNS), which can damage membrane lipids, structural and regulatory proteins, and DNA [37]. The results presented in this work showed that diacerhein treatment was effective in reducing the ROS and lipid peroxidation in the diaphragm muscle in *mdx* mice. These data also corroborate the reduction of muscle damage observed here, since studies show that oxidative stress precedes the degeneration process in *mdx* mice [39]. There are few studies focusing on the antioxidant effects of diacerhein, however, two studies point out that this drug modulates superoxide anion production from human neutrophils [33] and suppresses the increase in plasma nitric oxide (NO) levels during the development of rat adjuvant-induced arthritis [40]. In view of the promising antioxidant effects of diacerhein in the dystrophic muscle observed here, it is important that future studies better understand the mechanisms of the antioxidant action of this drug.

In addition to providing a comprehensive outcome assessment of treatment with diacerhein, the present study also compared the results obtained with diacerhein therapy to current glucocorticoid treatment widely used in DMD patients, with a main focus on the impact of prednisone. Glucocorticoid treatment is the only therapy proven to be beneficial in improving the skeletal and cardiac muscle function in Duchenne muscular dystrophy [27]. Our findings demonstrate that diacerhein treatment improves the dystrophic phenotype in the diaphragm muscle of *mdx* mice similar to prednisone therapy, indicated by the reduction in myonecrosis, oxidative stress state and inflammatory process.

In conclusion, this work suggests a potential use of diacerhein as an alternative drug in dystrophinopathy treatment and recommends that their anti-inflammatory and antioxidant properties in the dystrophic muscle should be better understood.

## Supporting information

S1 Fig Degeneration/regeneration process in dystrophic tibialis anterior muscle. Tibialis anterior (TA) cross-sections showing IgG staining (white arrow) in C57BL/10 (A), saline-treated *mdx* mice (B), prednisone-treated *mdx* mice (C) and diacerhein-treated *mdx* mice (D). The graphs (E) show the IgG staining (%) in the TA muscle of C57BL/10 mice (Ctrl), saline-treated *mdx* mice (*mdx*), prednisone-treated *mdx* mice (*mdx*P) and diacerhein-treated *mdx* mice (*mdx*D). TA cross-sections showing fibers which central nuclei (black arrow head) and peripheral nuclei (black arrow) in Ctrl (F), *mdx* (G), *mdx*P (H) and *mdx*D (I). The graphs show the nuclei central fibers (J) and peripheral nuclei fibers (K) in the TA muscle of Ctrl, *mdx*, *mdx*P and *mdx*D groups. ^a^*P* ≤ 0.05 compared with Ctrl mice, ^b^*P* ≤ 0.05 compared with *mdx* mice, ^c^*P* ≤ 0.05 compared with prednisone-treated *mdx* mice (one-way ANOVA with Tukey’s post-hoc test).
(TIF)

S2 Fig Inflammatory response morphology in dystrophic tibialis anterior muscle. Tibialis anterior (TA) cross-sections showing inflammatory area (outline) in C57BL/10 (A), saline-treated *mdx* mice (B), prednisone-treated *mdx* mice (C) and diacerhein-treated *mdx* mice (D). The graphs (E) show the inflammatory area (%) in the TA muscle of C57BL/10 mice (Ctrl), saline-treated *mdx* mice (*mdx*), prednisone-treated *mdx* mice (*mdx*P) and diacerhein-treated *mdx* mice (*mdx*D). TA cross-sections showing F4/80 staining (white arrow) in Ctrl (F), *mdx* (G), *mdx*P (H) and *mdx*D (I). In (J), graphs show the F4/80 staining (%) in the TA muscle of Ctrl, *mdx*, *mdx*P and *mdx*P groups. ^a^*P* ≤ 0.05 compared with Ctrl mice, ^b^*P* ≤ 0.05 compared with *mdx* mice (one-way ANOVA with Tukey’s post-hoc test).
(TIF)

S3 Fig Tumor necrosis factor alpha (TNF-α), and nuclear factor-kappa (NF-κB) levels in dystrophic tibialis anterior muscle. Western blotting analysis of tumor necrosis factor alpha (A, TNF-α), and nuclear factor-kappa (B, NF-κB). The blots of the proteins (top row) and of glyceraldehyde-3-phosphate dehydrogenase (loading control, bottom row), are shown. The graphs show protein levels in the crude extracts of tibialis anterior (TA) muscle from C57BL/10 mice (Ctrl), saline-treated *mdx* mice (*mdx*), prednisone-treated *mdx* mice (*mdx*P) and diacerhein-treated *mdx* mice (*mdx*D). The intensities of each band were quantified and normalized to those of the corresponding Ctrl. Relative values are expressed as mean ± standard deviation (SD). ^a^*P* ≤ 0.05 compared with Ctrl mice, ^b^*P* ≤ 0.05 compared with *mdx* mice, ^c^*P* ≤ 0.05 compared with prednisone-treated *mdx* mice (one-way ANOVA with Tukey’s post-hoc test).
(TIF)

S4 Fig Oxidative stress in dystrophic tibialis anterior muscle. Tibialis anterior (TA) cross-sections showing dihydroethidium (DHE) fluorescence in C57BL/10 (A), saline-treated *mdx* mice (B), prednisone-treated *mdx* mice (C) and diacerhein-treated *mdx* mice (D). The graphs (E) show the DHE staining area (%) in C57BL/10 mice (Ctrl), saline-treated *mdx* mice (*mdx*), prednisone-treated *mdx* mice (*mdx*P) and diacerhein-treated *mdx* mice (*mdx*D). TA cross-sections showing autofluorescent lipofuscin granules (white arrow) in Ctrl (F), *mdx* (G), *mdx*P (H) and *mdx*D (I). The graphs (J) show the number of lipofuscin granules x 10^−4^/mm^3^ in Ctrl, *mdx*, *mdx*P and *mdx*D groups. In (K), western blotting analysis of 4-hydroxynonenal (4-HNE)-protein adducts. Bands corresponding to protein (top row), and glyceraldehyde-3-phosphate dehydrogenase (GAPDH; used as loading control) (bottom row), are shown. The graphs show protein levels in the crude extracts of TA muscle from Ctrl, *mdx*, *mdx*P and *mdx*D groups. The intensities of each band were quantified and normalized to those of the corresponding Ctrl (in order to obtain relative values). All values expressed as mean ± standard deviation (SD). ^a^*P* ≤ 0.05 compared with Ctrl mice, ^b^*P* ≤ 0.05 compared with *mdx* mice (one-way ANOVA with Tukey’s post-hoc test).
(TIF)
