# Supplementary figures and images for: Dystrophic phenotype improvement in the diaphragm muscle of mdx mice by diacerhein
Source: PLoS One. 2017 Aug 7;12(8):e0182449. doi: 10.1371/journal.pone.0182449 (PMC5546703; doi:10.1371/journal.pone.0182449)

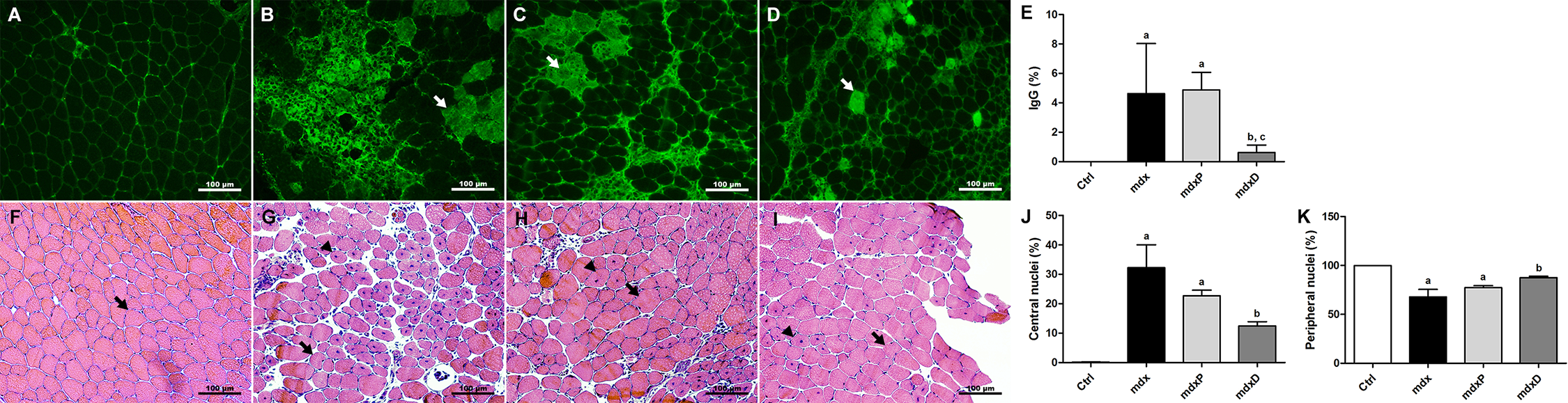

Supplement: S1 Fig — Tibialis anterior (TA) cross-sections showing IgG staining (white arrow) in C57BL/10 (A), saline-treated mdx mice (B), prednisone-treated mdx mice (C) and diacerhein-treated mdx mice (D). The graphs (E) show the IgG staining (%) in the TA muscle of C57BL/10 mice (Ctrl), saline-treated mdx mice (mdx), prednisone-treated mdx mice (mdxP) and diacerhein-treated mdx mice (mdxD). TA cross-sections showing fibers which central nuclei (black arrow head) and peripheral nuclei (black arrow) in Ctrl (F), mdx (G), mdxP (H) and mdxD (I). The graphs show the nuclei central fibers (J) and peripheral nuclei fibers (K) in the TA muscle of Ctrl, mdx, mdxP and mdxD groups. aP ≤ 0.05 compared with Ctrl mice, bP ≤ 0.05 compared with mdx mice, cP ≤ 0.05 compared with prednisone-treated mdx mice (one-way ANOVA with Tukey’s post-hoc test). (TIF) [file pone.0182449.s001.tif]

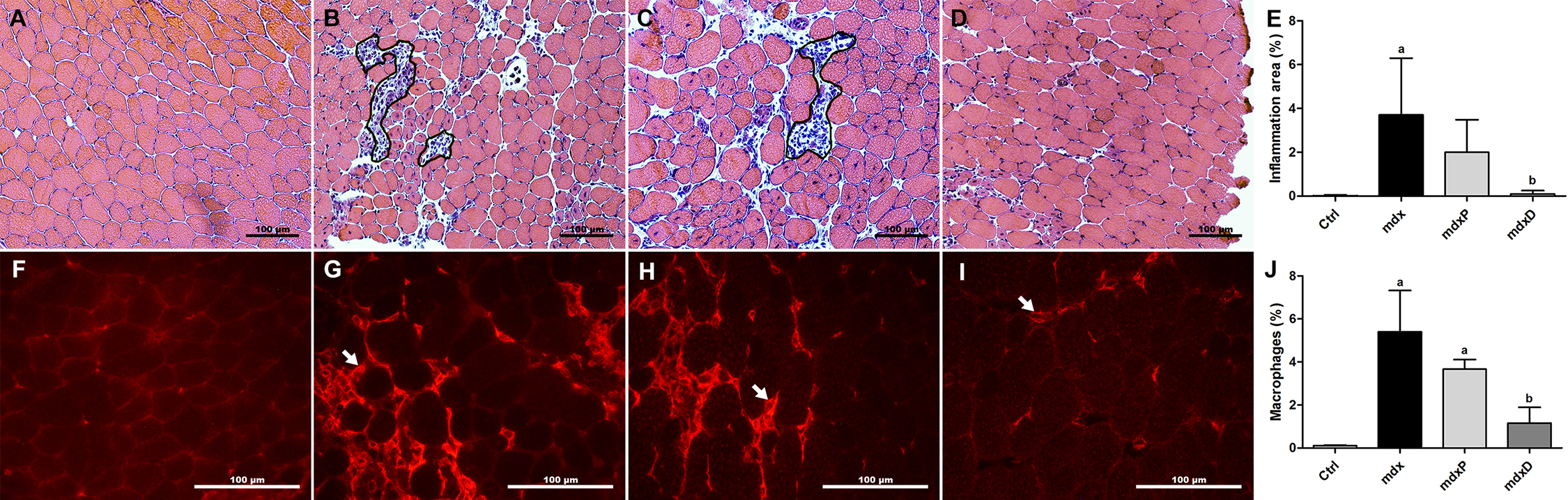

Supplement: S2 Fig — Tibialis anterior (TA) cross-sections showing inflammatory area (outline) in C57BL/10 (A), saline-treated mdx mice (B), prednisone-treated mdx mice (C) and diacerhein-treated mdx mice (D). The graphs (E) show the inflammatory area (%) in the TA muscle of C57BL/10 mice (Ctrl), saline-treated mdx mice (mdx), prednisone-treated mdx mice (mdxP) and diacerhein-treated mdx mice (mdxD). TA cross-sections showing F4/80 staining (white arrow) in Ctrl (F), mdx (G), mdxP (H) and mdxD (I). In (J), graphs show the F4/80 staining (%) in the TA muscle of Ctrl, mdx, mdxP and mdxP groups. aP ≤ 0.05 compared with Ctrl mice, bP ≤ 0.05 compared with mdx mice (one-way ANOVA with Tukey’s post-hoc test). (TIF) [file pone.0182449.s002.tif]

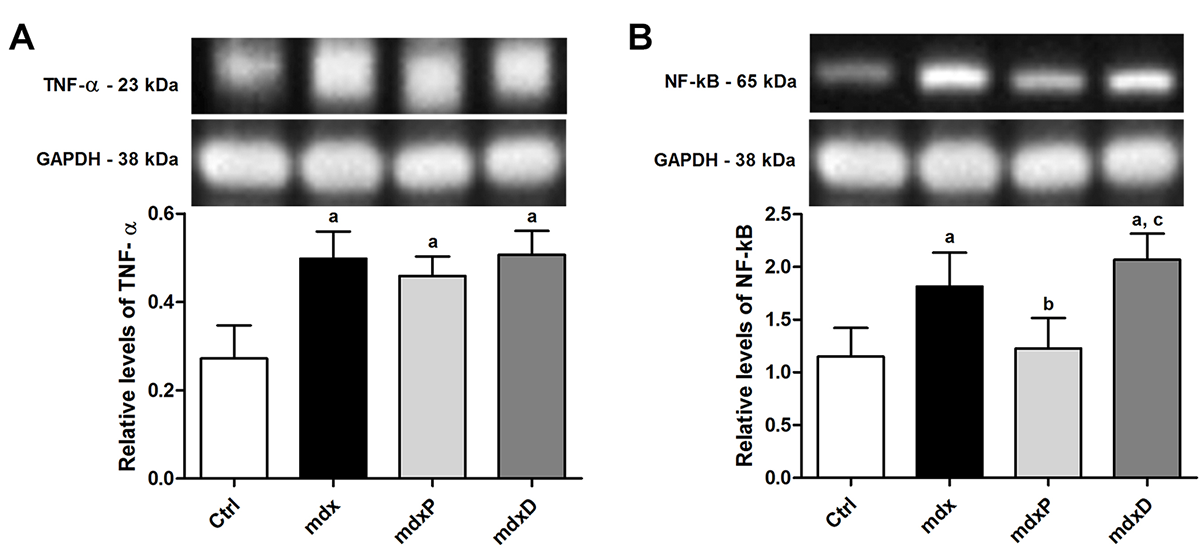

Supplement: S3 Fig — Western blotting analysis of tumor necrosis factor alpha (A, TNF-α), and nuclear factor-kappa (B, NF-κB). The blots of the proteins (top row) and of glyceraldehyde-3-phosphate dehydrogenase (loading control, bottom row), are shown. The graphs show protein levels in the crude extracts of tibialis anterior (TA) muscle from C57BL/10 mice (Ctrl), saline-treated mdx mice (mdx), prednisone-treated mdx mice (mdxP) and diacerhein-treated mdx mice (mdxD). The intensities of each band were quantified and normalized to those of the corresponding Ctrl. Relative values are expressed as mean ± standard deviation (SD). aP ≤ 0.05 compared with Ctrl mice, bP ≤ 0.05 compared with mdx mice, cP ≤ 0.05 compared with prednisone-treated mdx mice (one-way ANOVA with Tukey’s post-hoc test). (TIF) [file pone.0182449.s003.tif]

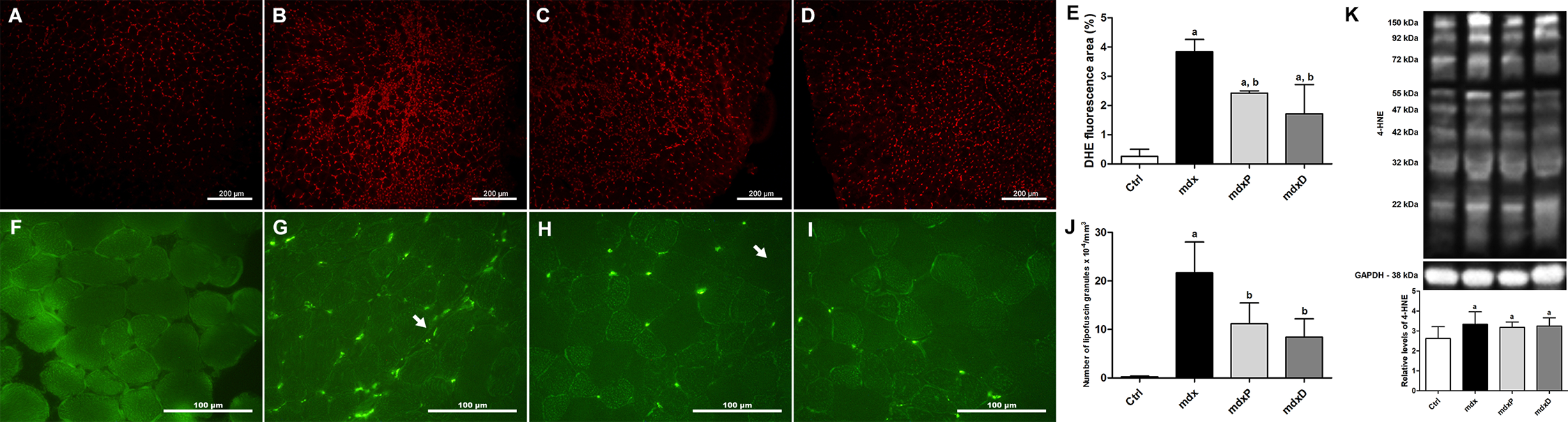

Supplement: S4 Fig — Tibialis anterior (TA) cross-sections showing dihydroethidium (DHE) fluorescence in C57BL/10 (A), saline-treated mdx mice (B), prednisone-treated mdx mice (C) and diacerhein-treated mdx mice (D). The graphs (E) show the DHE staining area (%) in C57BL/10 mice (Ctrl), saline-treated mdx mice (mdx), prednisone-treated mdx mice (mdxP) and diacerhein-treated mdx mice (mdxD). TA cross-sections showing autofluorescent lipofuscin granules (white arrow) in Ctrl (F), mdx (G), mdxP (H) and mdxD (I). The graphs (J) show the number of lipofuscin granules x 10−4/mm3 in Ctrl, mdx, mdxP and mdxD groups. In (K), western blotting analysis of 4-hydroxynonenal (4-HNE)-protein adducts. Bands corresponding to protein (top row), and glyceraldehyde-3-phosphate dehydrogenase (GAPDH; used as loading control) (bottom row), are shown. The graphs show protein levels in the crude extracts of TA muscle from Ctrl, mdx, mdxP and mdxD groups. The intensities of each band were quantified and normalized to those of the corresponding Ctrl (in order to obtain relative values). All values expressed as mean ± standard deviation (SD). aP ≤ 0.05 compared with Ctrl mice, bP ≤ 0.05 compared with mdx mice (one-way ANOVA with Tukey’s post-hoc test). (TIF) [file pone.0182449.s004.tif]
